# Evaluation of safety and efficacy of rapamycin-eluting balloon in patients with intracranial atherosclerotic stenosis: a cohort study

**DOI:** 10.1186/s13019-023-02204-6

**Published:** 2023-04-27

**Authors:** Guodong Xu, Xiaoli Dong, Yingying Tian, Liang Ma, Ning Han, Wentao Yao, Kuochang Yin, Nan Yin

**Affiliations:** Department of Neurology, Hebei General Hospital, Shijiazhuang, Hebei China

**Keywords:** Rapamycin-eluting balloon, Stenosis, Stroke, Angiography

## Abstract

**Objective:**

The safety and efficacy of drug-eluting balloon on the treatment of intracranial atherosclerotic stenosis (ICAS) remain unclear. Here, we present our observation in a cohort study on the safety and efficacy of rapamycin-eluting balloon for patients with ICAS.

**Methods:**

A total of 80 ICAS patients with stenosis degree of 70–99% were included. All patients were treated with rapamycin-eluting balloon and were followed up for 12 months after operation.

**Results:**

All patients were successfully treated, where the mean stenosis severity reduced from 85.1 ± 7.6 to 6 ± 4.9%. 8 patients experienced immediate post-operational complications. Two patients passed away during the first month of the follow-up period. Recurrent ischemic syndrome and angiographic restenosis only appeared 7 days after operation. During later follow-up period, none of the patients had clinical angiographic restenosis or needed target vessel revascularization.

**Conclusion:**

Our data suggest that intracranial stenting with rapamycin-eluting balloon seems to be safe and effective, although more clinical data are needed to support this notion.

## Introduction

Intracranial atherosclerotic stenosis (ICAS) is one of the important causes of ischemic stroke, which is more common in Asians than Caucasians [1]. In 2014, the incidence of intracranial atherosclerosis in patients with ischemic stroke or transient ischemic attack in China was 46.6%, where patients who had ICAS were accompanied by more severe symptoms, longer hospital stays, higher stroke recurrence rates, and higher recurrence rates as the degree of stenosis increased [2].

Usage of stenting for the treatment of ICAS has demonstrated some promising outcome over the past decade [3]. However, the restenosis rate in patients with symptomatic atherosclerotic lesions in the vertebral or intracranial arteries after stenting treatment was shown to be as high as 32% [4]. Drug-eluting balloon (DEB) employs a balloon-expandable stent system to release the carrying drugs into the vascular wall to suppress the proliferation and migration of endothelial and smooth muscle cells, thereby decreasing the ICAS [5]. Compared with stent implantation, the balloon-expandable stent system is more suitable for tortuous arteries, such as the cervical internal carotid artery, where it can pass through more easily to avoid atherosclerotic plaque damage. DEB has been extensively studied in coronary stenosis [6], however, systemic studies on its treatment of ICAS remains elusive.

In the present prospective cohort study, we applied rapamycin-eluting balloon to dilate the vascular stenosis of ICAS patients. Here, we present the clinical and follow-up results of 80 such patients.

## Methods

### Study design

This study was a prospective cohort study performed in the Department of Neurology, Hebei General Hospital from January 2017 to December 2021. The study was approved by the ethical committee of Hebei General Hospital and was performed according to the guidance of the Declaration of Helsinki. All patients or their guardians understood the study purpose and signed the written consent forms. All included patients were between the age of 40 and 80. All patients were diagnosed with symptomatic ICAS and the degree of stenosis of the affected arteries was between 70 and 99% based on intracranial angiography assessment. Stenosis vessels were in the internal carotid artery (intracranial segment), the middle cerebral artery, the basilar artery or the vertebral artery (intracranial segment) and its branches. Only ICAS with single lesion was included. All included patients demonstrated at least one risk factor for atherosclerotic plaque, including previous or existing hypertension, diabetes, hyperlipidemia, and smoking. All patients had a modified Rankin Score (mRS) ≤ 2 prior to enrollment. Only patients that were committed to a 1 year follow-up were included.

**Fig. 1:**
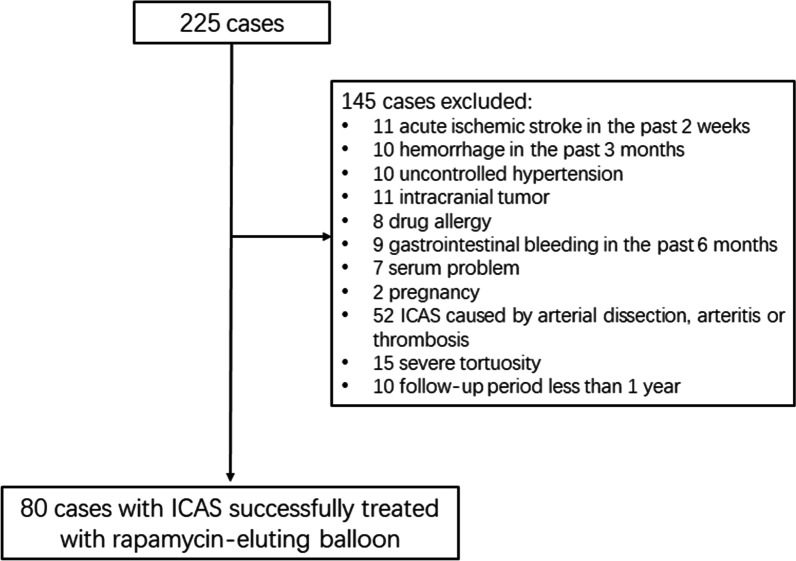
Flowchart of participant selection

Exclusion criteria include acute ischemic stroke in the past 2 weeks; intracranial hemorrhage, including cerebral parenchymal hemorrhage, massive subarachnoid hemorrhage and subdural/external hemorrhage in the past 3 months; hypertension (continuous systolic blood pressure ≥ 180 mmHg or diastolic blood pressure ≥ 110 mmHg) that could not be controlled by medication; presence of intracranial tumor, aneurysm or intracranial arteriovenous malformation; past stent implantation history on the target lesion; allergic to heparin, rapamycin, contrast agents, aspirin, clopidogrel, or anesthetics; history of gastrointestinal bleeding in the past 6 months; serum platelet level < 90 × 109/L; serum creatinine > 250 umol/L; International Normalized Ratio (INR) > 1.5; female patients who were pregnant or breastfeeding at the time of enrollment, or planning to become pregnant in the following year; life expectancy less than 1 year; patients that were involved in other ongoing clinical trials; ICAS caused by arterial dissection, arteritis or thrombosis based on radiography results; affected blood vessels were too tortuous for the equipment to reach the target position or to retrieve after completion. Detailed exclusion criteria were listed in Fig. 1.

### Operation

Medical history and characters obtained from general examination, blood test results and National Institutes of Health Stroke Scale (NIHSS) and modified Rankin Scale (mRS) scores were recorded for all patients. All patients received aspirin at a dose of 100 mg per day and clopidogrel at a dose of 75 g per day at least 5 days before the operation. On the day of operation, all patients were examined by computed tomography (CT)/CT perfusion (CTP) or magnetic resonance imaging (MRI)/MR perfusion (MRP), together with digital subtraction angiography (DSA). Angiographic stenosis % was calculated as previously described [7]. Rapamycin-eluting balloon was used for all patients. Operation status, equipment usage, complications arose during operation and operative images were recorded. Entire operation procedure was performed under local anesthesia. All patients were maintained on the same dose of aspirin and clopidogrel for 3 months after stenting.

A 6F guide catheter (Stryker Neurovascular) was placed into the cervical internal carotid artery (ICA) or vertebral artery (VA) at the C2 level via a transfemoral approach. Intervention was conducted through 0.35 mm coronary rapamycin-eluting balloon (Boston Scientific) and stent delivery system with soft-tipped hydrophilic wires (Boston Scientific). Wire manipulation and placement were based on patients’ feeling. Patients’ discomfort was monitored at all time. Angioplasty was achieved by slowly inflating an undersized coronary balloon (Boston Scientific) to its nominal pressure. Neurological assessment was performed if the discomfort became pronounced. All adverse complications were recorded.

### Follow-up

After operation, patients were monitored at the neurological intensive care unit to keep the systolic blood pressure under 140 mmHg. Antiplatelet drugs were prescribed for 1 year to the patients. Patients were followed up clinically and with DSA at 7 days, 1 month, 6 months and 12 months after operation. A follow-up angiogram was performed for all patients between 6 and 12 months after operation. Angiographic restenosis was defined as the presence of ≥ 50% stenosis in the stent region at any of the follow-up time point.

## Results

**Fig. 2:**
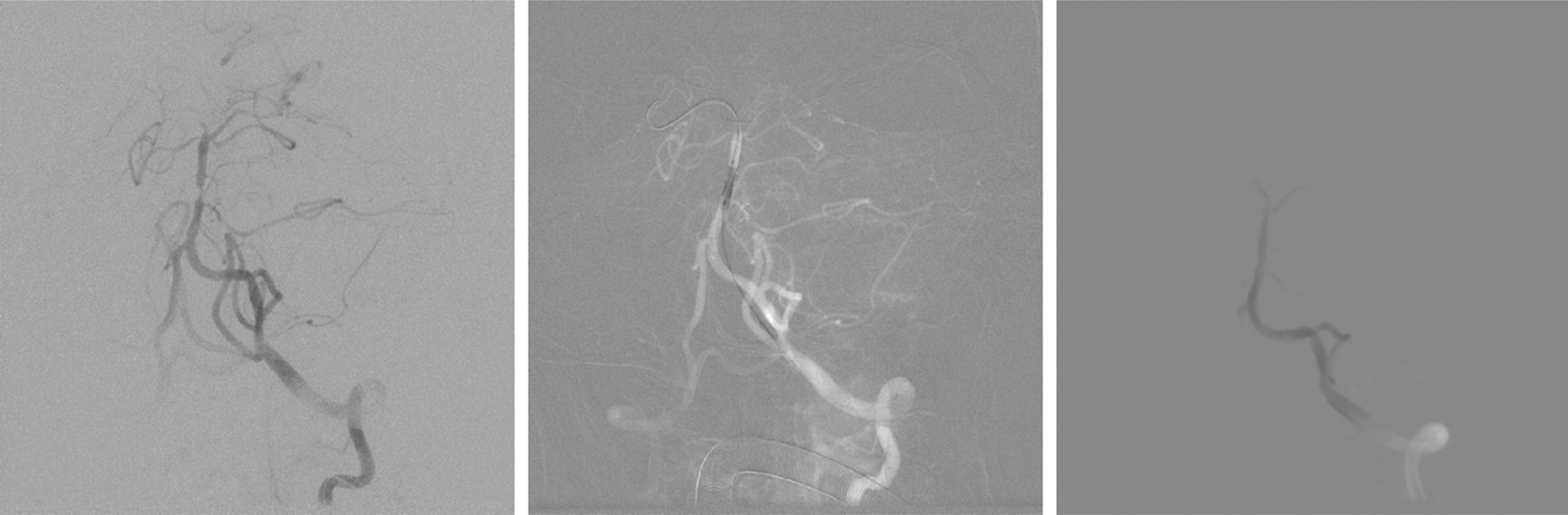
DSA images during the DEB operation

A total of 80 symptomatic ICAS patients treated with rapamycin-eluting balloon were included. Figure 2 illustrates the DSA images during the DEB process. All patients were successfully treated after the operation, where a reduction in stenosis to ≤ 50% luminal narrowing was achieved. Patients were between the age of 40–80 and 68.75% of them (55 out of 80) were male (Table 1). At the time of hospital admission, hypertension, peripheral vascular disease and coronary artery disease were the most common medical history of the patients, all of which appeared in over 50% of them (Table 1). The NIHSS score fell within the range of 13–38, which covered the moderate and severe stroke categories (Table 1). mRS score fell within the range of 2–5, covering slight, moderate, moderately severe and severe neurological disability caused by stroke (Table 1).

**Table 1:** Clinical characteristics recorded at hospital admission

Characteristic	N = 80
Age (mean ± SD)	59.4 ± 11.2
Sex (male:female)	55:25
Disease history (% of total)	
Hypertension	49 (61.25%)
Diabetes	38 (47.5%)
Hyperlipidemia	30 (37.5%)
Peripheral vascular disease	45 (56.25%)
Coronary artery disease	44 (55%)
NIHSS score (mean ± SD)	27.3 ± 8.1
mRS score (mean ± SD)	3.6 ± 1.1

Prior to operation, all patients experienced recurrent stroke (47 out of 80) and transient ischemic attack (TIA) (33 out of 80) (Table 2). Affected vessels included 33 ICA, 19 middle cerebral artery (MCA), 15 VA and 24 basilar artery (BA) (Table 2). The mean stenosis severity was reduced from 85.1 ± 7.6 to 6 ± 4.9% (Table 2). 8 patients demonstrated post-operational complications (Table 2), including 2 cases of hematoma at the puncture site, 2 cases of upper respiratory tract infection, 2 cases of gastric hemorrhage and 2 cases of angina pectoris.

**Table 2:** Clinical characteristics recorded before operation

Clinical details	N = 80
Recurrent clinical syndrome (% of total)	
Stroke	47 (58.75%)
Transient ischemic attack (TIA)	33 (41.25%)
Affected vessel (% of total)	
Internal carotid artery (ICA)	33 (41.25%)
Middle cerebral artery (MCA)	19 (23.75%)
Vertebral artery (VA)	15 (18.75%)
Basilar artery (BA)	24 (30%)
Angiographic stenosis (%)	85.1 ± 7.6%
Post-operation stenosis (%)	6 ± 4.9%
Presence of post-operational complications (yes:no)	8:72

Seven patients experienced recurrent cerebral ischemic syndrome 7 days after operation, including 6 cases of stroke and 1 case of TIA (Table 3). All syndrome had diminished since the 1 month follow-up time point and all patients were neurologically independent (Table 3). 1 patient passed away at the 7 days and 1 month follow-up time points, respectively (Table 3). Both of them were elderly patients in the group, 1 was 80 years old and 1 was 78 years old. Both of them suffered from angina pectoris complications after operation. 4 patients experienced angiographic restenosis 7 days after operation, but were all resolved at later time points (Table 3). 3 of them had stroke recurrence and 1 of them had TIA recurrence.

**Table 3:** Follow-up details

Clinical details	7 days (n = 79)	1 month (n = 78)	6 months (n = 78)	12 months (n = 78)
Recurrent clinical syndrome (% of total)				
Stroke	6 (7.5%)	0	0	0
Transient ischemic attack (TIA)	1 (1.25%)	0	0	0
Time to follow-up imaging (months)				8.7 ± 2.0
Mortality rate (% of total)	1 (1.25%)	1 (1.25%)	0	0
Angiographic restenosis (% of total)	4 (5%)	0	0	0

## Discussion

To the best of our knowledge, the present study is among the only a few studies that report the use of rapamycin-eluting balloon in the intracranial vasculature with clinical and follow-up results. We show that rapamycin-eluting balloon is a generally safe method, given that the recurrent ischemic syndrome rate, mortality rate and angiographic restenosis rate are all relatively low within a 12 month follow-up period. Based on a previous systemic review and meta-analysis, symptomatic ICAD patients treated with drug-eluting stent have 6.0% (95% CI 2.0–11.9%) of recurrent stroke/mortality rate within 30 days after operation [3]. The authors also report a significant difference between the severe [12.1% (95% CI 7.4–17.9%)] and moderate [1.4% (95% CI 0.2–3.8%)] patient groups [3]. Our rates of recurrent syndrome (0%) and mortality (2.5% 2 out of 80) are closer to the reporter moderate patient group, although most of our included patients suffered from severe stroke based on their NIHSS score.

Generally, DEB contains three components: an expandable stent platform that mechanically opens up the stenosed artery, a polymer coating that binds and releases drug and the drug itself. Therefore, it likely generates a mechanical and pharmacological “double effects” on the operated artery. Vessel toxicity is a theoretical risk for drug-eluting stent that can be caused by direct drug effects. However, a study that investigated the safety of drug-eluting stent in dog’s BA artery reveals no evidence of arterial or brain tissue [8]. In fact, the amount of drug released into the tissue from drug-eluting stent is negligible compared with the known toxic dose of rapamycin [9]. We have checked arterial and cerebral toxicity by angiography and clinical status, respectively and did not observe any sign of toxic effect. Another concern with drug-eluting stent is delayed endothelialization, which can manifest to stent thromboses even 6 months after treatment [10]. Nonetheless, it is not a commonly observed complication and can be prevented by prolonged antiplatelet treatment [10]. Therefore, we have applied 1 year antiplatelet treatment to all the included patients in this study and observed no such complications.

One limitation of the present study is its single-institutional nature, which only includes the Chinese patient population. Future multi-centered international studies are needed to verify our findings. The sample size was also relatively small.

## Conclusion

In summary, we observed that rapamycin-eluting balloon is a safe and effective treatment for ICAS, which needs to be further tested in randomized clinical trials conducted at the international level.

## Data Availability

Not applicable.

## References

[1] Kim JS, Bonovich D (2014). Research on intracranial atherosclerosis from the East and west: Why are the results different?. J Stroke.

[2] Wang Y, Zhao X, Liu L, Soo YO, Pu Y, Pan Y (2014). Prevalence and outcomes of symptomatic intracranial large artery stenoses and occlusions in China: the Chinese Intracranial Atherosclerosis (CICAS) Study. Stroke.

[3] Ye G, Yin X, Yang X, Wang J, Qi P, Lu J (2019). Efficacy and safety of drug-eluting stent for the intracranial atherosclerotic disease: a systematic review and meta-analysis. J Clin Neurosci.

[4] Investigators SS (2004). Stenting of symptomatic atherosclerotic lesions in the vertebral or intracranial arteries (SSYLVIA): study results. Stroke.

[5] Inoue T, Node K (2009). Molecular basis of restenosis and novel issues of drug-eluting stents. Circ J.

[6] Di Lorenzo E, Sauro R, Varricchio A, Capasso M, Lanzillo T, Manganelli F (2009). Benefits of drug-eluting stents as compared to bare metal stent in ST-segment elevation myocardial infarction: four year results of the PaclitAxel or Sirolimus-Eluting stent vs bare metal stent in primary angiOplasty (PASEO) randomized trial. Am Heart J.

[7] Abou-Chebl A, Bashir Q, Yadav JS (2005). Drug-eluting stents for the treatment of intracranial atherosclerosis: initial experience and midterm angiographic follow-up. Stroke.

[8] Levy EI, Hanel RA, Howington JU, Nemes B, Boulos AS, Tio FO (2004). Sirolimus-eluting stents in the canine cerebral vasculature: a prospective, randomized, blinded assessment of safety and vessel response. J Neurosurg.

[9] Holmes DR, Leon MB, Moses JW, Popma JJ, Cutlip D, Fitzgerald PJ (2004). Analysis of 1-year clinical outcomes in the SIRIUS trial: a randomized trial of a sirolimus-eluting stent versus a standard stent in patients at high risk for coronary restenosis. Circulation.

[10] Moreno R, Fernandez C, Hernandez R, Alfonso F, Angiolillo DJ, Sabate M (2005). Drug-eluting stent thrombosis: results from a pooled analysis including 10 randomized studies. J Am Coll Cardiol.

